# Development and characterization of novel microsatellite markers for *Tribolium castaneum* (Coleoptera: Tenebrionidae)

**DOI:** 10.1093/jisesa/ieag006

**Published:** 2026-02-02

**Authors:** Li Lim, Abdul Hafiz Ab Majid

**Affiliations:** Household & Structural Urban Entomology Laboratory, School of Biological Sciences, Universiti Sains Malaysia, Pulau Pinang; Household & Structural Urban Entomology Laboratory, School of Biological Sciences, Universiti Sains Malaysia, Pulau Pinang

**Keywords:** marker, store product pest, population genetics, next-generation sequencing

## Abstract

The red flour beetle, *Tribolium castaneum* (Herbst), is a major pest of stored products. Microsatellite markers offer valuable tools for population genetic studies; however, existing markers often exhibit limited polymorphism or lack validation in field-collected populations. In this study, microsatellite loci were identified from whole-genome sequencing data of *T. castaneum* and tested for their utility in Malaysian populations. A total of 13.8 million sequence reads yielded 108,318 primer pairs, from which 20 markers were selected for screening. Of these, 7 loci consistently amplified and exhibited high levels of polymorphism, producing 14 to 28 alleles per locus. Expected heterozygosity values ranged from 0.798 to 0.962, and polymorphic information content exceeded 0.750 for all loci, indicating their high informativeness. Population differentiation analysis revealed moderate genetic structuring among populations (mean *F_St_* = 0.162), suggesting restricted gene flow across sampled regions. These results demonstrate that the newly developed microsatellite markers are robust and highly polymorphic, providing a valuable resource for population genetic studies of *T. castaneum* and supporting efforts to understand pest dispersal and management in Malaysia.

## Introduction

The red flour beetle, *Tribolium castaneum* (Herbst) (Coleoptera: Tenebrionidae), is one of the most destructive pests of stored products, causing considerable economic losses in cereal-based commodities such as flour, grains, and processed foods (Attia et al. 2020, Chaubey 2023). Owing to its fully sequenced genome, short generation time, and ease of maintenance under laboratory conditions, it is also a well-established model organism for genetic and developmental research (Herndon et al. 2020, Klingler and Bucher 2022).

Molecular markers are widely used to investigate genetic variation, population structure, and dispersal patterns of pest species. Among them, microsatellites or simple sequence repeats are highly informative due to their abundance, co-dominant inheritance, and high polymorphism (Ijaz 2011, Zhong et al. 2022). They have been applied successfully in population genetics and pest management studies, offering insights into gene flow, resistance development, and invasion dynamics (Opadith et al. 2022, Neiva et al. 2023).

Microsatellite markers for *T. castaneum* have been developed previously, including genome-wide surveys and population-level studies. However, earlier efforts reported several drawbacks that limit their broad applicability. For example, many of the markers described by Demuth et al. (2007) exhibited amplification inconsistencies, deviations from Hardy-Weinberg equilibrium, or localization to genomic regions of low complexity. Subsequent work by Drury et al. (2009) applied a subset of these markers to population analyses, but the number of loci was limited, null alleles were frequent, and overall allelic diversity was relatively low. Moreover, validation was largely restricted to laboratory colonies or a limited set of global populations, with little attention to Southeast Asian populations where the pest is widespread.

Advances in next-generation sequencing (NGS) provide opportunities to overcome these limitations by enabling large-scale microsatellite mining from whole-genome data. This approach allows the development of more robust markers with improved polymorphism and greater applicability to diverse populations. This study applies NGS-based marker discovery to identify and validate new microsatellite loci for *T. castaneum*, with polymorphism tested across multiple field-collected populations in Malaysia. These markers expand the available genetic toolkit for *T. castaneum* and offer improved resolution for studies of population structure, invasion dynamics, and pest management.

## Materials and Methods

### Sample Collection and DNA Extraction

Samples were collected from locations with known infestations. The sampling sites are listed in Table 1. The samples were collected in January 2025, consisted of adult beetles, and were obtained from food warehouses and storage facilities. Specimens were collected using sticky traps and handpicking from rice grain stacks, with assistance from Pest Control Operators and the management of Padiberas Nasional Berhad. All samples were stored in 70% ethanol and kept at −20 °C before DNA extraction.

**Table 1.** Location of samples collected from Malaysia

State	District	Latitude	Longitude
**Sabah**	Kota Kinabalu	5°58′09.3″N	116°06′47.0″E
**Sabah**	Kota Kinabalu	6°06′26.4″N	116°12′09.2″E
**Sabah**	Kota Kinabalu	6°04′36.6″N	116°07′50.4″E
**Penang**	Prai	5°21′04.7″N	100°24′29.9″E
**Selangor**	Klang	3°01′06.4″N	101°22′06.8″E
**Selangor**	Klang	3°00′49.3″N	101°21′58.7″E
**Selangor**	Klang	3°00′25.6″N	101°22′29.2″E

One individual was randomly selected for whole-genome sequencing. Genomic DNA was extracted from the entire insect using the HiYield Plus Genomic DNA Mini Kit (Blood/Tissue/Cultured Cells) (Real Biotech Corp., Taipei, Taiwan) according to the manufacturer’s instructions. Approximately 50 µL of DNA was obtained and quantified using a NanoDrop 2000c spectrophotometer (Thermo Fisher Scientific, Massachusetts, United States). Illumina paired-end (PE) libraries with an average insert size of 150 bp were prepared following standard protocols (Illumina, San Diego, California, United States) and sequenced on the HiSeq platform. For marker validation, DNA was also extracted from 70 individuals (10 from each sampling site) to assess the polymorphism of the designed microsatellite markers.

### Microsatellite Marker Design

Microsatellite markers were identified from the genome sequence of *T. castaneum* using OSTRFPD v0.01 (Mathema et al. 2019), applying a minimum threshold of eight repeats for motifs ranging from di- to hexanucleotides. Primer pairs flanking the identified regions were designed with Primer3 (Untergasser et al. 2012) under default settings, with parameters set to a length of 17 to 26 bp, melting temperature of 58 to 63 °C, and GC content of 20% to 80%. Amplicon size was restricted to 100 to 150 bp, and primers predicted to form secondary structures were excluded.

### Microsatellite Marker Validation

Twenty microsatellite markers were selected from the designed set for polymorphism analysis based on repeat number and motif type. These markers were tested on DNA extracted from 70 individual *T. castaneum* specimens. PCR amplification was performed using a Thermal Cycler (TaKaRa, Japan) in a 25 µL reaction mixture containing 12.5 µL of Master Mix with green buffer (NX, Nuclix Biosolution, Malaysia), 0.5 µL each of 5 mM forward and reverse primers, 5 µL of DNA template, and nuclease-free water to volume. The PCR program consisted of an initial denaturation, followed by 40 cycles of denaturation at 94 °C for 30 s, annealing at locus-specific temperatures (Table 2) for 30 s, extension at 75 °C for 45 s, and a final extension at 75 °C for 5 min.

**Table 2.** Information about 20 microsatellites developed from *Tribolium castaneum*

Locus	Repeat motif	Primer sequences	^a^Tm (°C)	^b^ *N_A_*
**TCDi1**	(AG)_23_	F: GGATCCATATGATGTCTTTGTTGA R: AAATTCCTGTCAGCCGCA	53	28
**TCDi2**	(AC)_22_	F: CAGCCTCCACCCGATATAAG R: TGATTTCTTTGACACTGCCATT	54	1
**TCTri1**	(AAT)_32_	F: CGCTCAGTGAGAAGCCC R: CAATGTCTTCCACGATCCATT	54	14
**TCTri2**	(AAC)_13_	F: TTGATGGTCCTCCTTCATCA R: GGCATTACACTTGAAACGCAG	55	6
**TCTri3**	(ATC)_10_	F: GCCATTTATGTATGCCCTCG R: CTCATTTGTCGTCGTCTTCG	55	24
**TCTri4**	(AGC)_9_	F: ACACAGCCTACGCCTTTGAT R: GGATTATTCGGTGTCATCGG	55	8
**TCTri5**	(ACC)_13_	F: ATCCATGGTCAGAGCACCTC R: TGAGACGGAGGGACACATTT	58	0
**TCTri6**	(AAC)_13_	F: TTACAGCCCGGACAGCAC R: CGCTCGTTCCAGAGAATGTT	57	1
**TCTri7**	(AAC)_13_	F: TTCATCAATAAATAATTGTGTTTCGT R: GGCATTACACTTGAAACGCAG	49	3
**TCTri8**	(CAG)_11_	F: TGACTGGTGCAGGTGATGAT R: GGCGGTTTACGGTTATCCTAC	57	0
**TCTri9**	(ACC)_10_	F: GACGGGAAGGAATGGATGT R: GTCTACCCAACCGACCAGAG	57	0
**TCTri10**	(CCG)_9_	F: CCTGCTACCTCCTGGTCAAC R: ACAATTAGTGAAGGCAGCGG	57	1
**TCTetra1**	(AAAG)_8_	F: TCCTCATCCTCATTTCCGAC R: GAATGGCGATGGAGAGAGAA	56	20
**TCTetra2**	(AAAT)_8_	F: AGTGGGTCCGATTTGGTACG R: TGTAAGTTCGTGCTGTTCTAGGTT	57	22
**TCTetra3**	(AAAT)_9_	F: AACGCCGGGAATAATTTGA R: GGCAGCAGATTATTATTACCATCA	53	4
**TCTetra4**	(AAAT)_8_	F: AGAGCCGGTGTACACAACATT R: CAACGTGGAAGCTATACACAGG	57	15
**TCPenta1**	(ATTCC)_10_	F: TTCCACTCGTGCTGATTCC R: TCATCCGTAATGGAATGGAAA	53	0
**TCPenta2**	(AAAAT)_9_	F: GAGGCTTTGTGAGAATAATTTGG R: CCACCATGGCACATGTTTAC	53	25
**TCPenta3**	(AATGG)_8_	F: TGGAATGTAATGGAGAGTAAGGG R: ACTCGGGTTGATTCCATTCC	55	4
**TCPenta4**	(ATTCC)_8_	F: ATTCCATTCGGGTTGTTCC R: TAATGGAACGGAATGCAATG	52	0

a Tm (°C)—annealing temperature.

b *N_A_—*number of alleles observed.

Fragment analysis was conducted using the Fragment Analyzer Automated CE System (Agilent Technologies, California), and allele scoring was performed with ProSize 3.0 software (Agilent Technologies, California). Of the 20 markers tested, 7 were selected for further validation based on motif type and amplification quality. These consisted of one dinucleotide repeat (TCDi1), 2 trinucleotide repeats (TCTri1 and TCTri3), 3 tetranucleotide repeats (TCTetra1, TCTetra2, and TCTetra4), and 1 pentanucleotide repeat (TCPenta2). Mononucleotide repeats were excluded due to their high amplification error rate (Shinde et al. 2003, Baptiste et al. 2015).

Micro-Checker v2.2.0.3 (Van Oosterhout et al. 2004) was used to assess fragment analysis results, with a focus on detecting potential errors such as amplification failure, stuttering, or large allele dropout by estimating observed and expected null allele frequencies. Allele frequency parameters, including observed and expected heterozygosity, number of alleles, and PIC, were calculated using Cervus v3.0.7 (Kalinowski et al. 2007). Population differentiation (*F_St_*) was estimated according to Weir and Cockerham (1984) with GENEPOP on the web (Rousset 2008).

## Results and Discussion

Microsatellites were screened from 13,804,267 raw sequence reads. The identified repeats comprised 96.83% mononucleotides, 0.22% dinucleotides, 2.94% trinucleotides, 6.21 × 10^−3^% tetranucleotides, 1.64 × 10^−3^% pentanucleotides, and 5.17 × 10^−4^% hexanucleotides. From these, 108,318 primer pairs were designed, and the complete list is available in the Figshare repository (DOI: https://doi.org/10.6084/m9.figshare.30262639.v1).

From this set, 20 primers were selected for testing, most of which successfully genotyped 70 *T. castaneum* individuals from 7 populations, producing allele counts ranging from 0 to 28 (Table 2). Loci with *N_A_* values of 0 to 3 exhibited weak or inconsistent amplification and/or low polymorphism in field-collected populations and were therefore excluded from further analyses.

Seven markers were selected for further validation based on consistent amplification quality and high polymorphism, as indicated by high allele numbers (14 to 28 alleles per locus), expected heterozygosity, and PIC values across samples from 7 locations (Table 3). These values are higher than those reported in previous studies, where loci typically displayed 2 to 6 alleles (Drury et al. 2009) and demonstrate the utility of NGS-based marker development for increasing resolution in population genetic analyses.

**Table 3.** Characteristics of 7 microsatellite DNA loci developed for *Tribolium castaneum* and screened for a total of 70 specimens collected from 7 locations: results from software Micro-checker (stuttering, allele dropout, and present of null alleles), and software Cervus [number of alleles observed (*N_A_*), average expected (*H*_E_), and observed (*H*_O_) heterozygosities, and PIC value (PIC)]

Locus	Micro-checker			Cervus			
	Stuttering	Allele dropout	Presence of null alleles	*N_A_*	*H*_E_	*H*_O_	PIC
**TCDi1**	No	No	No	28	0.962	0.271	0.954
**TCTri1**	No	No	Yes	14	0.798	0.000	0.768
**TCTri3**	No	No	Yes	24	0.934	0.100	0.923
**TCTetra1**	No	No	No	20	0.911	0.714	0.897
**TCTetra2**	No	No	Yes	22	0.884	0.057	0.869
**TCTetra4**	No	No	Yes	15	0.887	0.071	0.870
**TCPenta2**	No	No	Yes	25	0.932	0.200	0.921

The validated loci revealed high levels of genetic diversity across populations. Expected heterozygosity (*H*_E_) ranged from 0.798 to 0.962, while PIC values were consistently high (>0.75) (Table 3), indicating that these markers are highly informative for genetic studies. However, several loci exhibited heterozygote deficiencies, as reflected in low observed heterozygosity (*H*_O_) and the presence of null alleles. As Micro-Checker analyses did not indicate amplification failure, stuttering, or allele dropout (Table 3), technical artefacts are unlikely to account for the observed heterozygote deficiencies. Instead, this pattern likely reflects the localized nature of infestations, where beetle populations are often confined within individual storage facilities, increasing the likelihood of inbreeding and population sub-structuring.

Population differentiation estimates based on *F*-statistics (Table 4) indicated moderate genetic structuring among populations (Duan et al. 2017, Seri Masran et al. 2019, Hall 2022), with an average *F_St_* of 0.162. Differentiation was particularly pronounced at loci such as TCTri3, TCTetra2, and TCPenta2, where values exceeded 0.20. These results suggest restricted gene flow among the sampled regions, possibly due to a limited natural dispersal ability of the beetles or barriers associated with storage and trade practices.

**Table 4.** Allele identity (*F* stats) for all populations of *Tribolium castaneum*

Locus	*F_Is_*	*F_St_*	*F_It_*
**TCDi1**	0.703	0.063	0.722
**TCTri1**	1.000	0.099	1.000
**TCTri3**	0.868	0.214	0.897
**TCTetra1**	0.075	0.173	0.235
**TCTetra2**	0.92	0.222	0.938
**TCTetra4**	0.907	0.153	0.922
**TCPenta2**	0.739	0.206	0.792
**All**	0.740	0.162	0.782

Populations from Sabah (East Malaysia) are geographically isolated from those in Peninsular Malaysia (Penang and Selangor), separated by the South China Sea, which naturally reduces opportunities for gene exchange. Even within Peninsular Malaysia, populations from northern (Penang) and central (Selangor) regions, there is also limited mixing of beetles between storage facilities that often operate independently. Similar to reports in other stored-product pests (Hernandez et al. 2015, Cordeiro et al. 2019), movement of *T. castaneum* between distant locations is more likely mediated by human transport through grain shipments than by natural dispersal. However, since grain movement within Malaysia is not uniform, genetic connectivity may remain restricted, leading to the observed structuring among populations.

## Data Availability

The primers designed in this study are deposited in the Figshare repository and publicly available at https://doi.org/10.6084/m9.figshare.30262639.v1
